# Location of residence associated with the likelihood of patient visit to the preoperative assessment clinic

**DOI:** 10.1186/1472-6963-6-13

**Published:** 2006-02-22

**Authors:** Judy E Seidel, Cynthia A Beck, Gaia Pocobelli, Jane B Lemaire, Jennifer M Bugar, Hude Quan, William A Ghali

**Affiliations:** 1 Department Community Health Sciences, University of Calgary, Health Sciences Centre, 3330 Hospital Drive NW, Calgary, Alberta, T2N 4N1, Canada; 2 Department of Medicine, University of Calgary, Foothills Hospital 1403-29^th ^Street NW Calgary Alberta, T2N 2T9, Canada; 3 Department of Psychiatry, University of Calgary, Foothills Hospital 1403-29^th ^Street NW Calgary Alberta, T2N 2T9, Canada; 4 Centre for Health and Policy Studies, University of Calgary, Health Sciences Centre, 3330 Hospital Drive NW, Calgary, Alberta, T2N 4N1, Canada; 5 Centre for Advancement of Health, Calgary Health Region, Foothills Hospital 1403-29^th ^Street NW Calgary Alberta, T2N 2T9, Canada

## Abstract

**Background:**

Outpatient preoperative assessment clinics were developed to provide an efficient assessment of surgical patients prior to surgery, and have demonstrated benefits to patients and the health care system. However, the centralization of preoperative assessment clinics may introduce geographical barriers to utilization that are dependent on where a patient lives with respect to the location of the preoperative assessment clinic.

**Methods:**

The association between geographical distance from a patient's place of residence to the preoperative assessment clinic, and the likelihood of a patient visit to the clinic prior to surgery, was assessed for all patients undergoing surgery at a tertiary health care centre in a major Canadian city. The odds of attending the preoperative clinic were adjusted for patient characteristics and clinical factors.

**Results:**

Patients were less likely to visit the preoperative assessment clinic prior to surgery as distance from the patient's place of residence to the clinic increased (adjusted OR = 0.52, 95% CI 0.44–0.63 for distances between 50–100 km, and OR = 0.26, 95% CI 0.21–0.31 for distances greater than 250 km). This 'distance decay' effect was remarkable for all surgical specialties.

**Conclusion:**

The present study demonstrates that the likelihood of a patient visiting the preoperative assessment clinic appears to depend on the geographical location of patients' residences. Patients who live closest to the clinic tend to be seen more often than patients who live in rural and remote areas. This observation may have implications for achieving the goals of equitable access, and optimal patient care and resource utilization in a single universal insurer health care system.

## Background

Prior to the development of preoperative assessment clinics, patients were typically admitted to the hospital before the day of surgery to undergo a medical evaluation by an anesthesiologist and when necessary, medical internists. The ultimate goal of the preoperative assessment clinic is to provide an efficient assessment of surgical patients prior to surgery. This is accomplished through a decrease in average costs associated with unnecessary laboratory tests, cancelled or delayed surgery, additional cost associated with intraoperative complications, and extended post-operative patient length of hospital stay [1-5]. Further, the preoperative assessment clinic assesses patient health status to optimize perioperative management, acts as a vehicle for patient education, and provides patients and their families with an opportunity to ask questions about the surgery [5,6].

The use of the preoperative assessment clinic is widespread and evidence of associated cost savings is growing. However, knowledge regarding whether patients have equitable access to these important clinics, based on where they live, is unknown. Most preoperative assessment clinics are located near or within a tertiary centre where the surgery takes place. Access could be compromised for patients who must travel long distances to receive centralized preoperative assessment clinic services. Several studies have examined geographical access to other medical services, such as cardiac procedures [7,8], breast cancer treatment [9], utilization of mammography [10], hospital discharge [11], and medical surgical care [12]. A decrease in the utilization of these medical services was observed for patients living longer distances from these services. It is possible that access to preoperative assessment clinics may also depend on where a patient lives, thereby leading to restricted access for those living further away.

In this study, we examined the association between geographical distance from a patient's residence to the preoperative assessment clinic located at a university-affiliated tertiary care centre, and the likelihood of a patient visit to this clinic prior to surgery.

## Methods

### Data sources

Hospital discharge data were used to identify all patients who underwent surgery at the Foothills Hospital, in Calgary, Alberta, between July 01, 1996 to March 31, 1998. Among these, we included for study all patients residing in the province of Alberta (whether inside or outside of Calgary city limits), as well as patients living in the neigbouring provinces of British Columbia and Saskatchewan, because those provinces typically refer some patients to the Foothills Hospital in Calgary for tertiary care services. Individuals who lived beyond these areas were excluded since they were residents of other provinces and unlikely to be typical geographically proximal tertiary care referrals; rather, we suspect that such cases represent individuals having surgery in Calgary because of personal ties to the city (e.g., friends or relatives residing in the city, in the context of permissible out-of-province surgery through Canada's inter-provincial portability of health insurance).

The surgical specialties of general surgery, cardiovascular/thoracic, gynecology, neurosurgery, orthopedics, plastic surgery, otolaryngology, urology, and oral surgery were included. Given the small number of patients in some specialties, the divisions of plastic surgery, otolaryngology, urology and oral surgery were combined into one group.

Discharge data were used to capture patient demographics, assessment and discharge dates, urgency of surgery (i.e. surgery that is necessary to mitigate what would otherwise be an imminently threatening medical condition), International Classification of Diseases, 9^th ^Edition, Clinical Modification (ICD-9-CM) diagnostic and procedure codes[13], most responsible surgical specialty, and patient postal codes. Those who underwent emergent surgery, day procedures, or in the rare case had surgery cancelled after admission because of patient or surgeon decision, were excluded. Cases with a missing procedure or postal code were also excluded.

This study was approved by the Conjoint Health Research Ethics Board of the University of Calgary.

### Variables

#### 1. Preoperative assessment clinic patient visit

Patients who attended the preoperative assessment clinic at the Foothills Medical Centre before their surgery were identified by linking the hospital discharge data with preoperative assessment clinic booking data. Patients were matched on last name and date of birth. Surgical assessment dates that were within 60 days of preoperative assessment clinic consultation dates were considered a match. Dates beyond 60 days would most likely be the result of something else and were therefore excluded to avoid misclassification. The preoperative assessment clinic is a multidisciplinary clinic that is staffed by nurses, anesthesiologists and internists; individual patients see one or more of these provider groups depending on their clinical profile. Typically patients who are scheduled to have surgery attend the preoperative clinic 1–2 weeks before their surgery date. Once their assessment is finished they return home and come back again for surgery. For the purpose of this study, focusing on geography and access, we assessed whether *any visit *to the preoperative clinic occurred. A more detailed description of visits to the preoperative clinic, by provider type, is reported in a recent paper by Bugar et al. [14].

#### 2. Confounding variables

Twenty-one patient comorbidities and procedures were identified through ICD-9 CM codes. The Deyo coding system [15] was applied to extract 17 comorbidities that comprise the Charlson comorbidity index, a measure of burden of comorbidity [16]. An additional two procedures and two comorbidities that may predict preoperative assessment clinic utilization were also included: previous percutaneous transluminal coronary angioplasty (ICD-9-CM code V458.2), previous coronary artery bypass grafting (V458.1), hypertension (401.xx – 405.xx), and unstable angina (411.1). Our selection of patient comorbidities and procedures for inclusion in this study were based on knowledge that these factors could alter a surgeon's decision as to whether they would refer the patient to the preoperative assessment clinic prior to surgery. For example, the more comorbidities that an individual patient has, the more likely the patient would be referred to the preoperative assessment clinic.

Two clinical reviewers examined all primary procedure codes independently using a predetermined classification scheme to determine whether the surgical procedure was major or minor [14]. Procedures involving brief anesthesia, limited tissue dissection, or anticipated short recovery period were considered minor. Discordant coding between reviewers was resolved by consensus [14].

#### 3. Measurement of distance

Distance from patient residence to preoperative assessment clinic was calculated by linking patient postal code to corresponding Canadian census enumeration areas using the Postal Code Conversion File [17]. The single link indicator (SLI), included in the Postal Code Conversion File, was used to establish a one-to-one relationship between a postal code and an enumeration area. The SLI identifies the geographic area with the majority of dwellings using the particular postal code. Straight-line distance between the geographical centroid of the census enumeration area for the patient and the preoperative assessment clinic was calculated using the latitude and longitude data contained in the Postal Code Conversion File. The following formula was used:

d = R arccos(sin(lat1)*sin(sin(lat2) + cos(lat1)*cos(lat2)*(lon1-lon2))

where, R is the radius of the earth, d=distance, lat=latitude, lon=longitude,

1 = patient residence, 2 = preoperative assessment clinic [18]

### Analysis

The likelihood of a patient visiting the preoperative assessment clinic before surgery was examined for each patient as a function of geographical distance from place of residence to preoperative assessment clinic. Graphical examination of the proportion of patients who visited the preoperative assessment clinic was undertaken using 50 km categories. Prior to categorizing distance, we examined distance as a continuous variable and noted that the relationship between distance and visit to the preoperative clinic was not linear and hence violated the linearity assumption required for treating distance as a continuous variable. Categorical data were thus analyzed with chi-square tests and analysis of variance was used to compare age across distance strata. Multiple logistic regression models were used to calculate the adjusted odds of a patient visit to the preoperative assessment clinic as a function of distance, while controlling for other covariates. In order to calculate the crude odds of a preoperative visit, only distance categories were entered into the model. The adjusted odds of a preoperative visit were calculated by forcing all distance categories into the model and adding age, sex, major versus minor surgery, and urgency of surgery and all 21 comorbidity variables. As recommended by Sun [19], backward elimination was undertaken to remove non-significant (p > 0.05 Wald test) covariates one at a time. The adjusted odds of a preoperative assessment clinic visit for only non-cardiac procedures were also calculated using the same modeling procedures. This analysis was undertaken because it was suspected that cardiac surgery referral practices in the region studied may differ from other surgical specialties [14]. We also conducted an analysis stratified by surgical division, to determine the adjusted odds of referral by distance category for each surgical division.

## Results

Between July 01, 1996 and March 31, 1998, 9506 patients underwent surgery at the study hospital and met the inclusion criteria. Of these, 5602 (58.9%) patients were referred and subsequently attended the hospital's preoperative assessment clinic before surgery. The mean and median straight-line distance from place of residence to preoperative assessment clinic was 55.1 km (95% CI 53.1–57.0) and 11.2 km, respectively. The shortest distance was 0 km, representing patients currently residing in long-term care at the study hospital, and the furthest distance was 878 km.

### Unadjusted utilization rates

**Figure 1:**
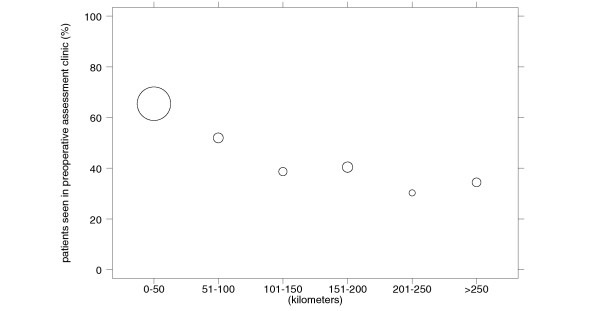
Proportion of surgical patients who visited the preoperative assessment clinic by distance. The size of each data point reflects the number of cases in each distance category.

Among patients who lived between 0 – 50 km from preoperative assessment clinic, 66% attended the clinic (Figure 1). The proportion of patients who attended the clinic decreased to 52% for those living between 51 – 100 km. A further reduction was observed for each consecutive 50 km increment, resulting in 39% for 101–150 km, 40% for 151–200 km, 30% for 201–250 km, and 34% for distances greater than 250 km.

### Clinical characteristics and risk factors

Table 1 summarizes the clinical characteristics of the 9506 patients by distance to preoperative assessment clinic. Patients living longer distances from the preoperative assessment clinic tended to be slightly older, male, and undergo more urgent surgery than those who lived closer. A higher proportion of neoplasms and heart disease comorbidities were also present at greater distances.

### Distance and adjusted utilization rates

The crude and adjusted odds ratios of a patient attending the preoperative assessment clinic before surgery are displayed in Table 2. The crude analysis demonstrates that patients were less likely to attend the preoperative assessment clinic prior to surgery as distance from the clinic increased. For example, patients who lived 50 km to 100 km from the clinic had approximately half the odds of being seen in the preoperative assessment clinic than did patients who lived closer (crude OR = 0.57; 95% CI 0.48–0.67), while patients who lived furthest from the clinic had approximately one-quarter the odds of being seen. Adjustment for differences in clinical factors, urgency of surgery, and whether the surgery was major or minor, had little effect on the odds ratio of attending the preoperative assessment clinic. After removing cardiac surgery cases from the multivariate model, because of suspected differences in referral practices, and adjusting for differences in the covariates above, there was little change in the odds of preoperative assessment clinic visits across distance categories.

### Distance and utilization by surgical specialty

Variation in the crude proportion of patients attending the preoperative assessment clinic was noted across surgical specialties and distance categories (Table 3). For all specialties, patients living within 50 km had the highest utilization rate (50% to 76%). A higher proportion of general surgery patients were seen in the clinic compared to the other surgery specialties for all distance categories except 151 to 200 km. After adjustment for differences in clinical factors (i.e. age, sex, and comorbidities), urgency of surgery, and whether the surgery was major or minor, the proportion of patients medically assessed in the clinic remained higher at closer distances to the preoperative assessment clinic for all surgical specialties (Table 4). It was noted that for cardiac surgery the 'distance decay' effect appeared at larger distances from the preoperative assessment clinic.

## Discussion

Our study demonstrates that the likelihood of a patient visiting the preoperative assessment clinic prior to surgery decreases as distance to the clinic increases. This 'distance decay' effect appears to persist after adjustment for clinical factors, surgical specialty, urgency of surgery, and whether the surgery was major or minor. Variation in utilization was also noted across surgical specialties and distance categories.

### Implications

The significance of these findings has implications for a considerable portion of the population who rely on health services in the regional tertiary care centre that we studied. The province of Alberta has a population of approximately 3 million. The two metropolitan health regions in the province (i.e. the Calgary Health Region and the Capital Health Region in and near Edmonton) provide health services to approximately 1 million people each, constituting 67% of the total provincial population. The remainder of the population (i.e. 1 million) lives outside of these two immediate metropolitan areas. In our study, 26% (N = 2512) of the 9506 surgical patients lived further than 50 kilometers from the Foothills Hospital, which is the main tertiary care facility in the Calgary Health Region.

A sizable literature exists on the importance of preoperative assessment and potential benefits to both patients and the health care system. Further, preoperative assessment is recognized as an important discipline in medicine. Previous studies have identified that clinical and other factors are important in the referral and utilization of preoperative assessment clinics. In a study by Bugar et al. [20], clinical factors were strongly associated with patient referral and utilization of the preoperative assessment clinic. Further, surgical specialty and type of clinic consultation were also factors in patient referral and utilization. For example the overall utilization rate of the preoperative assessment clinic for general surgery patients was 72%, while the consultation rate for this patient group was 19% for general internists and 39% for anesthesiologists, whereas overall utilization for neurosurgery was 63%, while the consultation rate for this patient group was 24% for general internists and 19% for anesthesiologists. Our study was designed to identify whether patient distance from the preoperative clinic was also an important factor, independent of such clinical factors. The results of our study suggest that patient distance from the clinic is indeed an important factor, and is in fact as important as clinical factors. Further, Table 1 displays how specific clinical factors play out by distance categories

Inequitable geographic access has significant implications given the identified and potential benefits of preoperative assessment for patients and the health system. For instance, decreased costs due to a reduction in laboratory testing, and a decrease in delayed or cancelled surgical procedures were reported benefits to the health systems in the United Kingdom and United States [1-3]. Although a decrease in clinical outcomes such as perioperative complications has not been substantiated as yet, studies have indicated that this is a potential benefit but one that is difficult to confirm [1,5]. Further, the preoperative assessment clinic provides patient-centred care. Patients and family members have an opportunity to discuss their concerns, medical risks, lessen anxiety, and obtain information about their surgery [5,20].

A second benefit of the preoperative assessment clinic is improved clinical documentation. Enhanced reporting of clinical information in patient charts can assist health professionals in making optimal perioperative management decisions. Good clinical documentation in records also provides the essential data needed for ICD-10 coding, costing, and billing [5,20]. Preoperative assessment clinics introduce a streamlined process for the patient and the health system through the use of standard assessment forms, provision of diagnostic services in one locale, and more complete medical records.

If we conclude that the inequitable access is problematic, what might be some of the solutions? One possibility is to ask all patients to travel for preoperative assessment clinic. This may or may not be acceptable to the general public because of personal, family, occupational, or financial reasons. Alternatively, satellite preoperative assessment clinics could be established, but this would obviously have staffing and health system cost implications. However, this may be a more appropriate option in a health system like Canada's that strives to achieve universal access, compared to the alternative above that would result in a shifting of costs from the health system to the patient. The burden of these extra costs on rural and remote residence can be significant, as demonstrated in an Australian study that examined the costs of accessing a surgical specialist [21]. Patients, who accessed a local as opposed to a metropolitan surgical specialist, were able to save an average of $1077 AU in out of pocket costs per specialist visit.

Another potential solution is the use of telemedicine technology. As telemedicine becomes more widespread, this alternative may be increasingly viable with time [22,23]. Once again this would have staffing, training, funding, and physician compensation implications.

Another 'remote triage' solution that is used in some settings is to have an anesthesiologist contact the patient at home by telephone to get a sense of whether a patient needs specialist consultation or specific tests prior to their surgery. Alternatively we could simply accept that preoperative assessment clinic assessment is not feasible for remote patients. However, some patients from remote regions might object to this status quo. This option is also of concern given that patients are being encouraged to participate in their own medical management as health care moves toward patient-centered care. Also, many surgeons might prefer or demand preoperative assessment clinic consultation prior to performing surgery. Patients could be seen immediately prior to surgery, eliminating an additional trip for the patient, and gaining some benefits such as patient centred care and documentation. However, the benefit of avoiding the cancellation of surgery would not be attainable. The development of referral guidelines to assist surgeons in deciding which patients should be sent to the preoperative clinic prior to surgery would also be helpful. The surgeon would be better prepared to identify and consult patients living in remote areas regarding the need for further medical assessment regardless of the patient's distance from the clinic.

Our initial study identifies a need for further inquiry into this complex referral and utilization process, to gain insight into stakeholder decision-making. For example, a survey would be helpful to identify the general public's willingness for extra travel, as well as objections to being "passed over" for preoperative assessment clinic. Surgeons could be asked about their willingness to forego preoperative assessment clinic or their tendency to simply skip preoperative assessment clinic for remote patients where they otherwise might refer them to preoperative assessment clinic. Consulting internists are also important stakeholders who could be questioned about their willingness to participate in satellite preoperative assessment clinics or work through telehealth.

### Study limitations

Our study has several limitations, the first of which is that we only studied one preoperative assessment clinic in a single health region in one province. Our findings may not apply to other health regions or provinces, although past studies have found similar distance decay effects for other health services [7-12]. Secondly, our study was undertaken in a single universal insurer health care system, and hence may not apply to other countries. However, studies from the United States and United Kingdom that examined geographical access also identified a decrease in utilization with increasing distance from the health service [7-12,24,25]. It is possible that patients at large distances from the preoperative assessment clinic received some form of preoperative assessment from their local physician or specialist outside of the clinic. Given that we used administrative date, we were limited for the most part in our ability to capture this information. However, the chance that preoperative assessment would have in fact taken place at the local level is likely low and would be atypical at this centre with the exception of patients scheduled for a cardiac procedure. Typically these patients see a cardiologist before attending the preoperative assessment clinic. Despite this however, we noted in our study that cardiac surgery patients did not appear to be as affected by distance as patients undergoing other surgical procedures.

There may also be other non-clinical confounders that we were unaware of and hence unable to capture or control for in this study that may have influenced referral to preoperative assessment clinic. For example, patients living in remote areas may have refused or were unable to travel to the preoperative assessment clinic.

Our use of straight-line distance to measure geographical access to the preoperative assessment clinic has some limitations. Research has shown that road network distance measures or travel times to a hospital more closely reflect 'true' distance because they take into account geographical and physical impeding structures such as roadways, mountains, rivers, etc. [26]. For these reasons, network and travel distances typically contain fewer errors and result in longer distance measures. As well, it should be noted that consideration of these features is likely less important in urban areas that are typically setup on a grid system, than in rural areas where geographic and physical structures are more prevalent [26]. However, the choice of whether to use a simple straight-line distance calculation versus network distance depends on the type of question under study. We were interested in the relative, rather than 'true' magnitude of distance and its effect on patient visits to the preoperative clinic. Further, our choice of distance measure was based on the assumption that straight-line distance is proportional to road network distance, as demonstrated in several studies [27,28]. It should also be noted that the use of centroids to approximate the location of patients introduces an additional source of error since these do not refer to the actual address of the patient. The amount of error introduced can vary depending on whether the patient lives in an urban or rural location [29,30].

Yet another caveat is that our study only examined *actual utilization *of the preoperative assessment clinic. Although it is possible that some of the referred patients may not have actually attended the clinic, the booking procedures for non-emergent surgery in the hospital studied are such that the vast majority of referrals actually lead to a clinic visit (– because planned surgical procedures are usually delayed or even cancelled if a patient does not attend the preoperative assessment clinic after a referral has occurred). On a final note, we grouped the specialties of urology, plastic, oral and otolaryngology surgery recognizing that relatively small number of surgical cases performed by each of these divisions would yield statistically unstable point estimates for the PAC clinic visit odds ratios that we present by division. By grouping these small divisions into a single combined grouping of "other" surgical divisions, we found that the relationship between patient visit and distance from the clinic still generally holds for these small surgical divisions.

## Conclusion

The present study demonstrates that the likelihood of a patient visit to the preoperative assessment clinic appears to depend on the geographical location of patients' residences. Patients who live closest to the clinic tend to visit the preoperative assessment clinic more often than patients who live in rural and remote areas. This observation may have implications for achieving the goal of equitable access for all patients, independent of where they live. Given the complexities of the referral and utilization process, further study into stakeholder decision-making is required to more fully understand this phenomenon.

## Competing interests

The author(s) declare they have no competing interests.

## Authors' contributions

JES was involved in study conception and design, performing the data analyses, interpretation of data and writing the initial manuscript. CAB and GP participated in study planning, data analyses and editing the manuscript. JMB, JBL, HQ were involved in the acquisition of the data and editing of the manuscript. WAG was involved in study conception and design, data analyses, interpretation of data and editing the manuscript. All authors read and approved the final manuscript.

## Pre-publication history

The pre-publication history for this paper can be accessed here:



## Figures and Tables

**Table 1:** Characteristics of patients undergoing surgery by distance categories

**Characteristic**	**0–50** **km** **(n = 6994)**	**51–100** **km** ** (n = 612)**	**101–150** **km** ** (n = 445)**	**151–200** **km** ** (n = 680)**	**201–250** **km** ** (n = 261)**	**>250** **km** ** (n = 514)**	**P**
age (mean in years)	52.7	54.7	54.9	54.5	55.7	54.6	<0.001
women (%)	59.1	60.1	52.1	47.2	43.3	49.0	<0.001
urgent assessment (%)	39.8	43.6	48.8	47.8	43.3	48.4	<0.001
major surgery (%)	61.2	61.8	62.9	64.1	65.5	64.2	0.35
chronic lung disease (%)	12.2	14.5	11.7	13.4	14.2	11.9	0.46
diabetes mellitus(%)	5.0	3.8	4.7	7.1	4.2	7.4	0.02
diabetes mellitus with complications (%)	1.2	1.5	2.3	1.8	0.4	1.4	0.25
mild liver disease (%)	0.4	0.5	0	0.3	1.9	0.2	0.003
moderate to severe liver disease (%)	0.2	0	0	0	0.4	0.4	0.34
neoplasm (%)	14.1	17.3	18.4	16.2	16.1	20.0	<0.001
metastatic solid tumor (%)	5.5	8.8	6.3	9.1	5.4	10.3	<0.001
hemiplegia (%)	0.7	0.8	1.1	0.9	0	0.6	0.58
chronic renal disease (%)	0.5	0.5	0.5	1.6	0.4	0.2	0.008
dementia (%)	0.1	0.3	0	0.2	0	0.2	0.74
rheumatologic disease (%)	1.8	1.8	2.0	2.2	1.2	0.8	0.49
peptic ulcer disease (%)	0.7	0.5	0.5	0.6	1.2	0.8	0.89
unstable angina (%)	2.3	2.1	4.7	3.4	2.7	4.7	0.001
hypertension (%)	23.0	25.2	28.3	28.1	23.4	24.5	0.009
congestive heart failure (%)	2.6	3.8	3.8	5.4	5.8	5.6	<0.001
recent myocardial infarction (%)	1.2	1.5	1.6	1.6	2.3	2.1	0.31
old myocardial infarction (%)	6.9	7.5	10.8	9.4	8.1	10.1	0.001
old percutaneous transluminal coronary angioplasty (%)	2.7	2.6	2.7	1.2	1.9	2.9	0.26
old coronary artery bypass graft (%)	1.8	1.6	1.6	2.4	0.8	1.8	0.70
cerebrovascular disease (%)	2.5	2.6	4.9	3.5	1.5	4.3	0.005
peripheral vascular disease (%)	3.4	3.3	2.9	4.9	4.6	4.3	0.26

**Table 2:** Odds ratios of preoperative assessment clinic utilization by distance compared with referent group (0–50 kilometers).

**Distance**	**Crude OR** **(95% Confidence Interval)**	Adjusted OR* (95% Confidence Interval)	**Adjusted OR non-CVT†** **(95% Confidence Interval)**
**0–50 km**	1.00 (reference)	1.00 (reference)	1.00 (reference)
**51–100 km**	0.57 (0.48–0.67)	0.52(0.44–0.63)	0.43 (0.36–0.53)
**101–150 km**	0.33 (0.27–0.40)	0.32 (0.26–0.39)	0.28 (0.22–0.36)
**151–200 km**	0.36 (0.30–0.42)	0.34 (0.29–0.40)	0.30 (0.25–0.37)
**201–250 km**	0.23 (0.17–0.30)	0.20 (0.15–0.26)	0.18 (0.13–0.26)
**>250 km**	0.27 (0.23–0.33)	0.26 (0.21–0.31)	0.20 (0.16–0.25)

*Adjusted for age, gender, urgency of surgery, major versus minor surgery, co-morbidities

† Adjusted for age, gender, urgency of surgery, major versus minor surgery, co-morbidities excluding cardio/thoracic surgical specialty.

**Table 3:** Crude proportion of preoperative assessment clinic utilization by surgical division and distance category (number referred/number operated)

**Surgical Division**	**0–50 kilometers**	**51–100 kilometers**	**101–150 kilometers**	**151–200 kilometers**	**201–250 kilometers**	**>250 kilometers**
**All divisions**	65.5% (4581/6994)	52.0% (318/612)	38.7% (172/445)	40.4% (275/680)	30.3% (79/261)	34.4% (177/514)
**General**	76.3% (1415/1854)	66.2% (100/151)	51.4% (37/72)	44.1%) (41/93)	45.7% (16/35)	51.3% (39/76)
**Cardio/thoracic**	49.6% (670/1351)	52.3% (67/128)	34.6% (45/130)	39.2% (91/232)	29.4% (32/109)	38.5% (62/161)
**Neurosurgery**	72.5% (665/917)	57.1% (48/84)	43.5% (47/108)	56.5% (87/154)	40% (18/45)	37.7% (35/93)
**Orthopedic**	59.8% (503/841)	52.4% (43/82)	50.0% (17/34)	30.6% (22/72)	27.6% (8/29)	26.3% (15/57)
**Obstetrics & Gynecology**	67.3% (922/1371)	38.8% (47/121)	29.2% (21/72)	25.0% (22/88)	16.0% (4/25)	21.1% (19/90)
Other*	61.5% (406/660)	28.3% (13/46)	17.2% (5/29)	29.3% (12/41)	5.6% (1/18)	18.9% (7/37)

*Other includes surgical specialties plastic, oral, otolaryngology and urology

**Table 4:** Adjusted odds ratio† of preoperative assessment clinic utilization by surgical division and distance compared with referent group (0 to 50 kilometers)

	**Odds Ratio (95% Confidence Interval)**					
**Surgical Specialty**	**0–50 kilometers**	**51–100 kilometers**	**101–150 kilometers**	**151–200 kilometers**	**201–250 kilometers**	**>250 kilometers**
**General**	1.00 (reference)	0.54 (0.37–0.79)	0.35 (0.21–0.57)	0.24 (0.15–0.38)	0.27 (0.13–0.56)	0.31 (0.19–0.51)
**Cardio/Thoracic**	1.00 (reference)	1.05 (0.71–1.55)	0.46 (0.31–0.69)	0.53 (0.39–0.72)	0.32 (0.20–0.51)	0.60 (0.42–0.86)
**Neurosurgery**	1.00 (reference)	0.48 (0.30–0.77)	0.26 (0.17–0.40)	0.48 (0.33–0.69)	0.23 (0.12–0.43)	0.23 (0.14–0.36)
**Orthopedic**	1.00 (reference)	0.82 (0.47–1.43)	0.72 (0.31–1.66)	0.24 (0.13–0.44)	0.20 (0.08–0.48)	0.17 (0.09–0.34)
**Obstetrics & Gynecology**	1.00 (reference)	0.22 (0.15–0.34)	0.18 (0.10–0.32)	0.19 (0.11–0.34)	0.07 (0.02–0.30)	0.13 (0.07–0.23)
Other*	1.00 (reference)	0.23 (0.12–0.47)	0.13 (0.04–0.35)	0.20 (0.09–0.42)	0.04 (0.01–0.30)	0.12 (0.05–0.30)

* Other includes plastic, oral, otolaryngology and urology surgical specialties

†Adjusted for age, gender, urgency of surgery, major versus minor surgery, co-morbidities
